# 1,5-Dimethyl-3-oxo-2-phenyl-2,3-dihydro-1*H*-pyrazol-4-aminium chloride–thio­urea (1/1)

**DOI:** 10.1107/S1600536811029989

**Published:** 2011-07-30

**Authors:** Shahzad Murtaza, Muhammad Hamza, M. Nawaz Tahir

**Affiliations:** a University of Gujrat, Department of Chemistry, Hafiz Hayat Campus, Gujrat, Pakistan; b University of Sargodha, Department of Physics, Sargodha, Pakistan

## Abstract

In the title compound, C_11_H_14_N_3_O^+^·Cl^−^·CH_4_N_2_S, the components are connected into a two-dimensional polymeric structure parallel to (001) *via* N—H⋯Cl, N—H⋯O, N—H⋯S and C—H⋯S hydrogen bonds. The dihedral angle between the phenyl and 2,3-dihydro-1*H*-pyrazole rings is 44.96 (7)°.

## Related literature

For the structure of 1,5-dimethyl-3-oxo-2-phenyl-2,3-dihydro-1*H*-pyrazol- 4-aminium 2-hy­droxy­benzoate, see: Chitradevi *et al.* (2009 ▶).
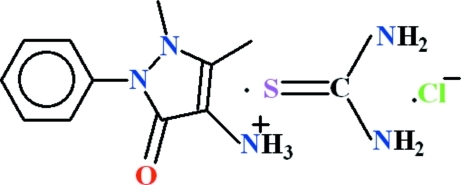


## Experimental

### 

#### Crystal data

C_11_H_14_N_3_O^+^·Cl^−^·CH_4_N_2_S
*M* *_r_* = 315.82
Monoclinic, 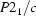

*a* = 9.9733 (11) Å
*b* = 8.2572 (8) Å
*c* = 18.859 (2) Å
β = 90.851 (4)°
*V* = 1552.9 (3) Å^3^
*Z* = 4
Mo *K*α radiation
μ = 0.38 mm^−1^
*T* = 296 K
0.30 × 0.15 × 0.14 mm

#### Data collection

Bruker Kappa APEXII CCD diffractometer
Absorption correction: multi-scan (*SADABS*; Bruker, 2005 ▶) *T*_min_ = 0.935, *T*_max_ = 0.950
14413 measured reflections
3876 independent reflections
2948 reflections with *I* > 2σ(*I*)
*R*_int_ = 0.032

#### Refinement

*R*[*F* ^2^ > 2σ(*F* ^2^)] = 0.039
*wR*(*F* ^2^) = 0.111
*S* = 1.02
3876 reflections
192 parameters
H atoms treated by a mixture of independent and constrained refinement
Δρ_max_ = 0.28 e Å^−3^
Δρ_min_ = −0.30 e Å^−3^

### 

Data collection: *APEX2* (Bruker, 2009 ▶); cell refinement: *SAINT* (Bruker, 2009 ▶); data reduction: *SAINT*; program(s) used to solve structure: *SHELXS97* (Sheldrick, 2008 ▶); program(s) used to refine structure: *SHELXL97* (Sheldrick, 2008 ▶); molecular graphics: *ORTEP-3 for Windows* (Farrugia, 1997 ▶) and *PLATON* (Spek, 2009 ▶); software used to prepare material for publication: *WinGX* (Farrugia, 1999 ▶) and *PLATON*.

## Supplementary Material

Crystal structure: contains datablock(s) global, I. DOI: 10.1107/S1600536811029989/gk2395sup1.cif

Structure factors: contains datablock(s) I. DOI: 10.1107/S1600536811029989/gk2395Isup2.hkl

Supplementary material file. DOI: 10.1107/S1600536811029989/gk2395Isup3.cml

Additional supplementary materials:  crystallographic information; 3D view; checkCIF report

## Figures and Tables

**Table 1:** Hydrogen-bond geometry (Å, °)

*D*—H⋯*A*	*D*—H	H⋯*A*	*D*⋯*A*	*D*—H⋯*A*
N3—H3*A*⋯S1^i^	0.92 (2)	2.28 (2)	3.1619 (17)	159.9 (19)
N3—H3*B*⋯O1^ii^	0.90 (2)	1.87 (2)	2.764 (2)	174 (2)
N3—H3*C*⋯Cl1^iii^	0.952 (19)	2.08 (2)	3.0316 (16)	180 (2)
N4—H4*A*⋯Cl1	0.86	2.41	3.2404 (17)	163
N4—H4*B*⋯O1^iv^	0.86	2.12	2.970 (2)	170
N5—H5*A*⋯Cl1	0.86	2.74	3.4956 (19)	148
N5—H5*A*⋯S1^v^	0.86	2.87	3.3768 (17)	120
N5—H5*B*⋯Cl1^vi^	0.86	2.56	3.4091 (18)	171
C10—H10*B*⋯S1^vi^	0.96	2.85	3.505 (2)	126

Symmetry codes: (i) 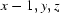; (ii) 
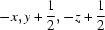
; (iii) 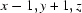; (iv) 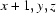; (v) 
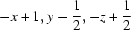
; (vi) 
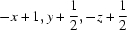
.

**Fig. 1.**
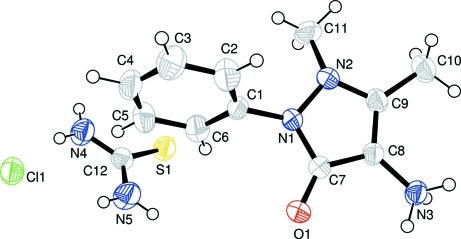
View of the title compound with the atom numbering scheme. The displacement ellipsoids are drawn at the 50% probability level. H-atoms are shown as small spheres of arbitrary radii.

**Fig. 2.**
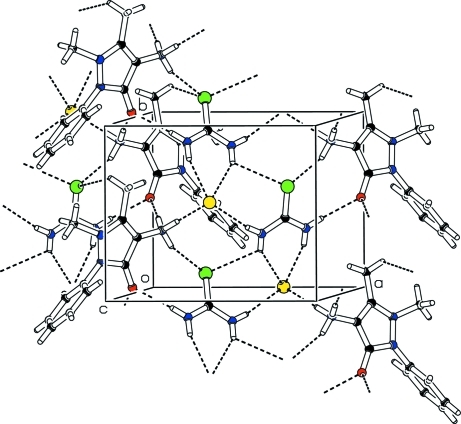
The partial packing (PLATON; Spek, 2009) showing hydrogen-bond interactions.

## supplementary crystallographic information

### Comment

The crystal structure of 1,5-dimethyl-3-oxo-2-phenyl-2,3-dihydro-1*H*-pyrazol- 4-aminium 2-hydroxybenzoate (Chitradevi *et al.*, 2009) has been published which is related to the title compound (Fig. 1).

The asymmetric unit of title compound consists of three components: 1,5-dimethyl-3-oxo-2-phenyl-2,3-dihydro-1*H*-pyrazol-4-aminium cation, chloride ion and thiourea molecule. In cation the phenyl ring A (C1—C6) and 2,3-dihydro-1*H*-pyrazole ring B (N1/N2/C7/C8/C9) are planar with r. m. s. deviations of 0.005 and 0.020 Å. The dihedral angle between A/B is 44.96 (7)°. The attached atoms O1, N3, C10 and C11 are at a distance of -0.122 (3), 0.005 (3), 0.034 (3) and 0.513 (3) Å respectively, from the mean plane of B. The thiourea molecule (S1/C12/N4/N5) is planar with r.m.s. deviations of 0.003 Å. There exist intermolecular hydrogen bonds of N–H···Cl, N–H···O, N–H···S and C–H···S types (Table 1, Fig. 2). The crystal components are connected by hydrogen bonds into infinite two dimensional polymeric network parallel to (0 0 1)

### Experimental

4-Aminophenazone (0.203 g, 1.0 mmol) and thiourea (0.076 g, 1.0 mmol) were dissolved in ethanol (15 ml) and the mixture was acidified by 1 N HCl. The mixture was refluxed for one hour and solvent was evaporated on rotary evaporator to almost dryness. The crude product was recrystallized from ethanol yielding light yellow needles of the title compound.

### Refinement

The coordinates of of NH_3 _group H atoms were refined. Other H atoms were positioned geometrically (N—H = 0.86, C–H = 0.93–0.96 Å) and were included in the refinement in the riding model approximation, with *U*_iso_(H) = x*U*_eq_(C, N), where x = 1.5 for CH_3_ and NH_3_ and x = 1.2 for other H-atoms.

### Crystal data

**Table:**

C_11_H_14_N_3_O^+^·Cl^−^·CH_4_N_2_S	*F*(000) = 664
*M**_r_* = 315.82	*D*_x_ = 1.351 Mg m^−^^3^
Monoclinic, *P*2_1_/*c*	Mo *K*α radiation, λ = 0.71073 Å
Hall symbol: -P 2ybc	Cell parameters from 2948 reflections
*a* = 9.9733 (11) Å	θ = 2.0–28.4°
*b* = 8.2572 (8) Å	µ = 0.38 mm^−^^1^
*c* = 18.859 (2) Å	*T* = 296 K
β = 90.851 (4)°	Needle, light yellow
*V* = 1552.9 (3) Å^3^	0.30 × 0.15 × 0.14 mm
*Z* = 4	

### Data collection

**Table:**

Bruker Kappa APEXII CCD diffractometer	3876 independent reflections
Radiation source: fine-focus sealed tube	2948 reflections with *I* > 2σ(*I*)
graphite	*R*_int_ = 0.032
Detector resolution: 7.50 pixels mm^-1^	θ_max_ = 28.4°, θ_min_ = 2.0°
ω scans	*h* = −13→12
Absorption correction: multi-scan (*SADABS*; Bruker, 2005)	*k* = −10→6
*T*_min_ = 0.935, *T*_max_ = 0.950	*l* = −24→25
14413 measured reflections	

### Refinement

**Table:**

Refinement on *F*^2^	Primary atom site location: structure-invariant direct methods
Least-squares matrix: full	Secondary atom site location: difference Fourier map
*R*[*F*^2^ > 2σ(*F*^2^)] = 0.039	Hydrogen site location: inferred from neighbouring sites
*wR*(*F*^2^) = 0.111	H atoms treated by a mixture of independent and constrained refinement
*S* = 1.02	*w* = 1/[σ^2^(*F*_o_^2^) + (0.0519*P*)^2^ + 0.4062*P*] where *P* = (*F*_o_^2^ + 2*F*_c_^2^)/3
3876 reflections	(Δ/σ)_max_ < 0.001
192 parameters	Δρ_max_ = 0.28 e Å^−^^3^
0 restraints	Δρ_min_ = −0.30 e Å^−^^3^

### Special details

**Table:**

Geometry. Bond distances, angles *etc*. have been calculated using the rounded fractional coordinates. All su's are estimated from the variances of the (full) variance-covariance matrix. The cell e.s.d.'s are taken into account in the estimation of distances, angles and torsion angles
Refinement. Refinement of *F*^2^ against ALL reflections. The weighted *R*-factor *wR* and goodness of fit *S* are based on *F*^2^, conventional *R*-factors *R* are based on *F*, with *F* set to zero for negative *F*^2^. The threshold expression of *F*^2^ > σ(*F*^2^) is used only for calculating *R*-factors(gt) *etc*. and is not relevant to the choice of reflections for refinement. *R*-factors based on *F*^2^ are statistically about twice as large as those based on *F*, and *R*- factors based on ALL data will be even larger.

### Fractional atomic coordinates and isotropic or equivalent isotropic displacement parameters (Å^2^)

**Table:**

	*x*	*y*	*z*	*U*_iso_*/*U*_eq_	
O1	0.02683 (12)	0.52568 (13)	0.17891 (7)	0.0429 (4)	
N1	0.15623 (14)	0.67708 (15)	0.10210 (8)	0.0380 (4)	
N2	0.15254 (14)	0.83360 (15)	0.07471 (8)	0.0395 (5)	
N3	−0.10897 (15)	0.84880 (18)	0.20387 (9)	0.0369 (5)	
C1	0.22930 (16)	0.55157 (19)	0.06724 (9)	0.0365 (5)	
C2	0.2008 (2)	0.5159 (2)	−0.00289 (11)	0.0539 (7)	
C3	0.2738 (2)	0.3964 (3)	−0.03623 (12)	0.0631 (8)	
C4	0.3709 (2)	0.3132 (2)	0.00038 (13)	0.0568 (7)	
C5	0.3968 (2)	0.3477 (3)	0.07011 (13)	0.0587 (7)	
C6	0.3260 (2)	0.4689 (2)	0.10411 (11)	0.0488 (6)	
C7	0.05681 (16)	0.65825 (18)	0.15067 (9)	0.0335 (5)	
C8	−0.00154 (15)	0.81326 (18)	0.15630 (9)	0.0328 (4)	
C9	0.05869 (16)	0.91713 (19)	0.11086 (9)	0.0360 (5)	
C10	0.0318 (2)	1.0926 (2)	0.09755 (12)	0.0506 (6)	
C11	0.2742 (2)	0.9003 (2)	0.04478 (12)	0.0528 (7)	
S1	0.70196 (5)	0.59619 (6)	0.27908 (3)	0.0490 (2)	
N4	0.76836 (16)	0.35228 (19)	0.19593 (9)	0.0531 (6)	
N5	0.57194 (16)	0.3262 (2)	0.25376 (10)	0.0579 (6)	
C12	0.67972 (17)	0.4139 (2)	0.24001 (10)	0.0398 (5)	
Cl1	0.64868 (5)	0.01267 (5)	0.13661 (3)	0.0476 (2)	
H2	0.13349	0.57146	−0.02737	0.0647*	
H3	0.25677	0.37259	−0.08371	0.0758*	
H3A	−0.148 (2)	0.757 (3)	0.2222 (10)	0.0554*	
H3B	−0.084 (2)	0.913 (3)	0.2403 (12)	0.0554*	
H3C	−0.185 (2)	0.900 (2)	0.1825 (11)	0.0554*	
H4	0.41951	0.23278	−0.02225	0.0681*	
H5	0.46218	0.28957	0.09489	0.0704*	
H6	0.34432	0.49342	0.15140	0.0586*	
H10A	0.00647	1.10791	0.04870	0.0759*	
H10B	0.11123	1.15417	0.10810	0.0759*	
H10C	−0.03966	1.12835	0.12730	0.0759*	
H11A	0.34012	0.91528	0.08178	0.0791*	
H11B	0.25437	1.00266	0.02288	0.0791*	
H11C	0.30826	0.82695	0.00990	0.0791*	
H4A	0.75487	0.25846	0.17735	0.0637*	
H4B	0.83942	0.40608	0.18589	0.0637*	
H5A	0.56147	0.23283	0.23429	0.0695*	
H5B	0.51243	0.36276	0.28217	0.0695*	

### Atomic displacement parameters (Å^2^)

**Table:**

	*U*^11^	*U*^22^	*U*^33^	*U*^12^	*U*^13^	*U*^23^
O1	0.0447 (7)	0.0331 (6)	0.0512 (7)	0.0023 (5)	0.0121 (6)	0.0107 (5)
N1	0.0424 (8)	0.0269 (7)	0.0450 (8)	0.0047 (5)	0.0125 (7)	0.0051 (6)
N2	0.0432 (8)	0.0291 (7)	0.0466 (9)	0.0016 (6)	0.0112 (7)	0.0070 (6)
N3	0.0362 (8)	0.0326 (7)	0.0421 (9)	0.0020 (6)	0.0066 (6)	−0.0039 (6)
C1	0.0348 (8)	0.0302 (7)	0.0449 (10)	0.0017 (6)	0.0116 (7)	0.0002 (7)
C2	0.0556 (12)	0.0522 (11)	0.0538 (12)	0.0118 (9)	−0.0051 (10)	−0.0050 (9)
C3	0.0764 (15)	0.0585 (13)	0.0547 (13)	0.0047 (11)	0.0069 (11)	−0.0174 (10)
C4	0.0596 (12)	0.0352 (9)	0.0763 (15)	0.0044 (9)	0.0263 (11)	−0.0066 (9)
C5	0.0512 (12)	0.0495 (11)	0.0755 (15)	0.0192 (9)	0.0085 (11)	0.0085 (10)
C6	0.0506 (11)	0.0476 (10)	0.0483 (11)	0.0100 (8)	0.0030 (9)	0.0026 (8)
C7	0.0335 (8)	0.0326 (8)	0.0343 (8)	0.0014 (6)	0.0021 (7)	0.0020 (6)
C8	0.0318 (8)	0.0305 (7)	0.0362 (8)	0.0016 (6)	0.0025 (7)	−0.0008 (6)
C9	0.0373 (9)	0.0298 (8)	0.0410 (9)	0.0020 (6)	0.0008 (7)	0.0006 (7)
C10	0.0546 (11)	0.0319 (9)	0.0655 (13)	0.0052 (8)	0.0041 (10)	0.0057 (8)
C11	0.0569 (12)	0.0411 (10)	0.0610 (13)	−0.0050 (8)	0.0257 (10)	0.0052 (9)
S1	0.0444 (3)	0.0403 (3)	0.0627 (3)	−0.0045 (2)	0.0134 (2)	−0.0056 (2)
N4	0.0495 (9)	0.0461 (9)	0.0641 (11)	−0.0048 (7)	0.0135 (8)	−0.0113 (8)
N5	0.0479 (9)	0.0522 (10)	0.0740 (12)	−0.0143 (8)	0.0133 (9)	−0.0110 (9)
C12	0.0366 (9)	0.0402 (9)	0.0426 (10)	−0.0004 (7)	−0.0018 (7)	0.0038 (7)
Cl1	0.0445 (3)	0.0443 (3)	0.0542 (3)	0.0060 (2)	0.0050 (2)	−0.0028 (2)

### Geometric parameters (Å, °)

**Table:**

S1—C12	1.6891 (18)	C2—C3	1.383 (3)
O1—C7	1.2554 (19)	C3—C4	1.366 (3)
N1—C1	1.432 (2)	C4—C5	1.366 (3)
N1—C7	1.369 (2)	C5—C6	1.387 (3)
N1—N2	1.3921 (18)	C7—C8	1.411 (2)
N2—C9	1.355 (2)	C8—C9	1.359 (2)
N2—C11	1.454 (2)	C9—C10	1.494 (2)
N3—C8	1.438 (2)	C2—H2	0.9300
N3—H3B	0.90 (2)	C3—H3	0.9300
N3—H3A	0.92 (2)	C4—H4	0.9300
N3—H3C	0.952 (19)	C5—H5	0.9300
N4—C12	1.324 (2)	C6—H6	0.9300
N5—C12	1.325 (2)	C10—H10A	0.9600
N4—H4A	0.8600	C10—H10B	0.9600
N4—H4B	0.8600	C10—H10C	0.9600
N5—H5B	0.8600	C11—H11A	0.9600
N5—H5A	0.8600	C11—H11B	0.9600
C1—C2	1.380 (3)	C11—H11C	0.9600
C1—C6	1.364 (3)		
			
N2—N1—C1	120.81 (14)	C7—C8—C9	109.79 (14)
N2—N1—C7	109.78 (12)	N3—C8—C9	127.24 (14)
C1—N1—C7	127.13 (13)	N2—C9—C8	108.13 (14)
N1—N2—C9	107.49 (13)	C8—C9—C10	129.66 (16)
N1—N2—C11	118.56 (13)	N2—C9—C10	122.19 (15)
C9—N2—C11	126.10 (13)	C1—C2—H2	120.00
H3B—N3—H3C	105.7 (18)	C3—C2—H2	120.00
C8—N3—H3C	115.0 (13)	C2—C3—H3	120.00
C8—N3—H3A	112.9 (14)	C4—C3—H3	120.00
C8—N3—H3B	113.3 (13)	C5—C4—H4	120.00
H3A—N3—H3B	108.2 (19)	C3—C4—H4	120.00
H3A—N3—H3C	100.7 (17)	C4—C5—H5	120.00
H4A—N4—H4B	120.00	C6—C5—H5	120.00
C12—N4—H4A	120.00	C5—C6—H6	120.00
C12—N4—H4B	120.00	C1—C6—H6	120.00
H5A—N5—H5B	120.00	H10B—C10—H10C	109.00
C12—N5—H5A	120.00	C9—C10—H10C	109.00
C12—N5—H5B	120.00	C9—C10—H10A	109.00
N1—C1—C6	119.27 (16)	C9—C10—H10B	109.00
C2—C1—C6	121.05 (16)	H10A—C10—H10B	110.00
N1—C1—C2	119.68 (15)	H10A—C10—H10C	109.00
C1—C2—C3	119.05 (18)	N2—C11—H11B	109.00
C2—C3—C4	120.2 (2)	N2—C11—H11A	109.00
C3—C4—C5	120.3 (2)	H11A—C11—H11C	110.00
C4—C5—C6	120.3 (2)	N2—C11—H11C	109.00
C1—C6—C5	119.10 (19)	H11A—C11—H11B	110.00
N1—C7—C8	104.54 (13)	H11B—C11—H11C	109.00
O1—C7—C8	131.16 (15)	N4—C12—N5	117.65 (16)
O1—C7—N1	124.22 (14)	S1—C12—N4	122.12 (13)
N3—C8—C7	122.97 (14)	S1—C12—N5	120.22 (14)
			
C1—N1—N2—C9	169.48 (14)	N1—C1—C2—C3	178.85 (17)
C1—N1—N2—C11	−39.5 (2)	C6—C1—C2—C3	−1.3 (3)
C7—N1—N2—C9	5.48 (18)	C2—C1—C6—C5	0.3 (3)
C7—N1—N2—C11	156.51 (16)	N1—C1—C6—C5	−179.83 (17)
N2—N1—C1—C2	−56.2 (2)	C1—C2—C3—C4	1.2 (3)
C7—N1—C1—C2	104.8 (2)	C2—C3—C4—C5	−0.2 (3)
N2—N1—C1—C6	123.92 (17)	C3—C4—C5—C6	−0.9 (3)
C7—N1—C1—C6	−75.1 (2)	C4—C5—C6—C1	0.8 (3)
N2—N1—C7—C8	−4.53 (18)	O1—C7—C8—N3	5.1 (3)
N2—N1—C7—O1	172.53 (15)	O1—C7—C8—C9	−174.75 (18)
C1—N1—C7—O1	9.8 (3)	N1—C7—C8—N3	−178.19 (15)
C1—N1—C7—C8	−167.26 (15)	N1—C7—C8—C9	2.02 (19)
N1—N2—C9—C10	177.29 (16)	N3—C8—C9—C10	0.0 (3)
N1—N2—C9—C8	−4.06 (18)	C7—C8—C9—N2	1.30 (19)
C11—N2—C9—C8	−152.29 (17)	C7—C8—C9—C10	179.82 (18)
C11—N2—C9—C10	29.1 (3)	N3—C8—C9—N2	−178.49 (16)

### Hydrogen-bond geometry (Å, °)

**Table:**

*D*—H···*A*	*D*—H	H···*A*	*D*···*A*	*D*—H···*A*
N3—H3A···S1^i^	0.92 (2)	2.28 (2)	3.1619 (17)	159.9 (19)
N3—H3B···O1^ii^	0.90 (2)	1.87 (2)	2.764 (2)	174 (2)
N3—H3C···Cl1^iii^	0.952 (19)	2.08 (2)	3.0316 (16)	180 (2)
N4—H4A···Cl1	0.86	2.41	3.2404 (17)	163
N4—H4B···O1^iv^	0.86	2.12	2.970 (2)	170
N5—H5A···Cl1	0.86	2.74	3.4956 (19)	148
N5—H5A···S1^v^	0.86	2.87	3.3768 (17)	120
N5—H5B···Cl1^vi^	0.86	2.56	3.4091 (18)	171
C10—H10B···S1^vi^	0.96	2.85	3.505 (2)	126

Symmetry codes: (i) *x*−1, *y*, *z*; (ii) −*x*, *y*+1/2, −*z*+1/2; (iii) *x*−1, *y*+1, *z*; (iv) *x*+1, *y*, *z*; (v) −*x*+1, *y*−1/2, −*z*+1/2; (vi) −*x*+1, *y*+1/2, −*z*+1/2.
